# Evaluating participant experience in Balint online sessions held during the COVID-19 pandemic – lessons learnt and moving forward

**DOI:** 10.1192/bjo.2021.678

**Published:** 2021-06-18

**Authors:** Nikhita Handa, Romy Garbutt, Sylvia Chudley

**Affiliations:** 1 East Lancashire Hospitals NHS Trust; 2 The Balint Society

## Abstract

**Aims:**

From the outset of the COVID-19 global pandemic and the lockdown that subsequently ensued, a challenge was posed to reshape previously face-to-face meetings in all walks of life. One area that rose to this, with quick introduction of online sessions, was the Balint Group. We aimed to take a snapshot of the effect virtual Balint sessions have had and analyse the themes that members of virtual Balint groups have been identifying about their online group experience at this particularly challenging time for healthcare workers. We hope this will inform both leaders and participants of future online groups of the benefits and pitfalls found by these members reflecting on their first experiences of virtual Balint.

**Method:**

Seven members of virtual Balint groups across the UK were randomly selected for interview from a pool of volunteers facilitated by the UK Balint Society after the first 6 months of their first virtual Balint experience. Interviews were conducted by two academic foundation doctors who were not members of the Balint groups. Qualitative thematic analysis was then conducted on these interview transcripts. Going forward, as Balint groups continue online, the researchers plan to interview further group members and leaders to look for change and development in the primary themes identified.

**Result:**

Key positive themes identified when discussing virtual Balint were ease of access, increased anonymity, attention to facial expressions and interaction with participants from different parts of the country. The most common drawback themes were a lack of socialising and different group dynamic as well as the expected technical and environmental challenges. Interestingly all participants reported that ‘silence’ and ‘sitting/stepping back’ were still used in their online sessions. Core theme analysis indicates the virtual Balint descriptions draw out sentiments of safe, open and structured sessions. In these early sessions a frequent theme was the increased role of the leader.

**Conclusion:**

All participants interviewed so far have felt their online experiences have had many positive aspects. They highlight areas they feel virtual Balint could develop to better replicate the original sessions. The fact some interviewees would prefer to maintain online Balint groups even when ‘in person’ options resume makes it likely this will not be a transient rise in virtual Balint and that the style may be here to stay. Based on this, the role for feedback and constant evaluation and improvement will be central to virtual Balint evolution.

